# Internet-based interventions for disordered gamblers: study protocol for a randomized controlled trial of online self-directed cognitive-behavioural motivational therapy

**DOI:** 10.1186/1471-2458-13-10

**Published:** 2013-01-08

**Authors:** David C Hodgins, Gordon H Fick, Robert Murray, John A Cunningham

**Affiliations:** 1 Departments of Psychology and Psychiatry, University of Calgary, Calgary, Canada; 2 Department of Community Health Sciences, University of Calgary, Calgary, Canada; 3 Centre for Addiction and Mental Health, Toronto, Canada; 4 Department of Psychology and the Dalla Lana School of Public Health, University of Toronto, Toronto, Canada

**Keywords:** Clinical trial, Brief intervention, Gambling disorders, Online intervention, Trial protocol

## Abstract

**Background:**

Gambling disorders affect about one percent of adults. Effective treatments are available but only a small proportion of affected individuals will choose to attend formal treatment. As a result, self-directed treatments have also been developed and found effective. Self-directed treatments provide individuals with information and support to initiate a recovery program without attending formal treatment. In previous research we developed an telephone-based intervention package that helps people to be motivated to tackle their gambling problem and to use basic behavioral and cognitive change strategies. The present study will investigate the efficacy of this self-directed intervention offered as a free online resource. The Internet is an excellent modality in which to offer self-directed treatment for gambling problems. The Internet is increasingly accessible to members of the public and is frequently used to access health-related information. Online gambling sites are also becoming more popular gambling platforms.

**Method/Design:**

A randomized clinical trial (N=180) will be conducted in which individuals with gambling problems who are not interested in attending formal treatment are randomly assigned to have access to an online self-directed intervention or to a comparison condition. The comparison condition will be an alternative website that offers a self-assessment of gambling involvement and gambling-related problems. The participant’s use of the resources and their gambling involvement (days of gambling, dollars loss) and their gambling problems will be tracked for a twelve month follow-up period.

**Discussion:**

The results of this research will be important for informing policy-makers who are developing treatment systems.

**Trial registration:**

ISRCTN06220098

## Background

### Significance

Gambling disorders are broadly defined as persistent and recurrent gambling that disrupts personal, family or vocational functioning [1]. Prevalence rates vary among jurisdictions according to availability and accessibility of gambling as well as definitional, measurement and other research design factors [2,3]. In Canada, all provinces have conducted one or more prevalence surveys and rates of problem gambling range from 0.7 to 1.4% of adults [4]. The personal, social, and economic costs of gambling disorders are large – including impairment or loss of relationships, stress-related medical problems, elevated risk of suicide, criminal offences and financial difficulties [3]. In addition, rates of comorbid mental health disorders such as mood, anxiety, attention deficit and substance use disorders are higher than expected [5,6].

Development and provision of effective treatment for gambling disorders is imperative. However, only about one in ten gamblers with a lifetime diagnosis of gambling disorder will ever seek treatment [7,8]. Many of these problem gamblers are unwilling to access treatment, often because of stigma, embarrassment or a desire to handle their problems on their own [9-12]. These problem gamblers, nonetheless, can be helped. Research has demonstrated the effectiveness of self-directed interventions for gambling problems [13-15]. This area deserves more attention because it addresses a cost-effective means of helping problem gamblers without requiring them to come to treatment. The aim of self-directed interventions is to help problem gamblers where they are, thus circumventing many of the barriers associated with traditional treatment.

#### Treatment outcome in disordered gambling

Outcome research for problem gambling treatment is limited but independent groups of investigators have begun to establish an evidence base in two related areas: cognitive-behavioral models [16-21] and brief self-directed treatments [22]. Brief treatments involve provision of self-directed written materials or limited contact with clients. For individuals not willing to seek formal treatment, brief interventions may be an attractive and effective, non-threatening alternative [23]. In two separate randomized clinical trials (N= 102, N = 314), we recruited individuals suffering gambling problems who wanted self-directed treatment but were not willing to attend formal treatment. Both trials supported the efficacy of a cognitive-behavioural self-help workbook sent via mail combined with a motivational telephone intervention [13-15]. The workbook was developed based upon earlier qualitative research on the process of recovery from pathological gambling [24,25]. It was designed to be brief, easy to read and to include practical change strategies [25]. It has been translated from English into a number of languages (French, Japanese, Portuguese, German, Swedish, and Norwegian) and is disseminated in a number of jurisdictions (e.g., Iowa, Oregon, Alberta). In the Hodgins et al. (2009) trial, participants at follow-up specifically identified using the workbook’s cognitive and behavioral strategies and viewed them as important in their recovery. A logical extension of this type of treatment is the use of the Internet as a platform for providing this type of self-directed treatment. Three factors support this direction: Internet access is widespread, the Internet is used to provide health treatment in a variety of areas and Internet-based gambling continues to grow.

#### The internet as a treatment platform

The use of the Internet as a platform has begun to be exploited in areas other than problem gambling. In the larger area of eHealth interventions, Portnoy and colleagues [26] reviewed 75 randomized controlled trials of different health behaviours and found general support for computer-based interventions. Despite the promise of the Internet, there remains limited research done in this area in regard to disordered gambling [23]. In Sweden, a randomized trial investigated the efficacy of a therapist-assisted web-based cognitive-behavioral program [27]. Compared with pathological gamblers on a waitlist, participants receiving the intervention had better gambling outcomes at three months. These gains were maintained over a 36 month follow-up interval. Cooper [28] and Wood [29,30] conducted qualitative studies investigating the use of online support groups among gamblers.

Our research team is also undertaking a randomized controlled trial of a brief personalized feedback intervention for problem gamblers (CYG; http://www.CheckYourGambling.net). In the CYG, the participant completes a brief assessment and then receives a personalized feedback report. Our pilot data (N = 61) indicated a decrease in gambling expenditures among individuals who volunteered to use the Internet self-assessment program [31], although our larger trial indicated that this effect is modest [32].

Such personalized feedback is often regarded as the first step in a self-directed brief intervention. However, there have been no reports of the development and evaluation of full Internet-based interventions that incorporate the full range of cognitive behavioural exercises that have been successful in paper-based self-directed formats. Problem gamblers have certainly indicated an interest in Internet-based services, with half of possible/probable pathological gamblers voicing an interest in a recent Ontario general population survey (N = 8467) [33]. The goal of this proposed research is to establish whether self-directed materials placed in an online context can provide a new tool to help problem gamblers deal with their concerns.

#### Access to the internet is crucial

The growing availability of the Internet makes it an ideal vehicle to help improve the accessibility of services for problem gamblers. Internet use has become widespread and is increasing. In 2011, 82% of Canadians had access to the Internet [34] and the majority of Internet users has accessed a health website [35]. Internet users include individuals with gambling disorders. A general population survey found that 73% of problem gamblers had home access to the Internet in Ontario in 2007 [36]. As our history of radio, TV and VCR ownership demonstrates, any remaining disparities in current use of the Internet across groups will likely reduce to the point that access is close to universal.

#### The growth of internet gambling

The first Internet gambling sites opened in 1995 and in 2011, there was an estimated 2400 sites operated by 650 different companies [37]. Estimates of participation rates in Canada range from 2.1% to 3.5% of adults [38], which are surprisingly high given that these companies are essentially operating illegally. Recently some provincial governments in Canada are beginning to offer legalized online gambling which will increase participation.

### Potential moderators and mediators of brief Internet interventions

How do brief interventions work and for whom? There has been little research into potential moderators and mediators of brief treatments in gambling disorders. In terms of moderators, exploratory analyses of our recent self-directed treatment for gambling problems (Hodgins et al. 2009) uncovered two factors that reliably predicted outcome- previous gambling or mental health treatment experience and self-efficacy. Participants who had no previous treatment and those that rated themselves as more likely to be successful had better outcomes regardless of the level of gambling problem severity. It is possible that these individuals are “natural” self-changers or, alternatively, that treatment-seeking is an indicator of one dimension of problem severity. Self-efficacy is often identified as a predictor of outcome from substance addictions [39,40] and may predict better success with self-help methods [41]. Self-efficacy can also act as a mediator of outcome. In our recent trial we found that improvement in self-efficacy as well as baseline self-efficacy correlated with improvement in gambling involvement [15]. In the smoking area, a study that compared an Internet-based cognitive-behavioural intervention to an information only control similarly found that self-efficacy was a significant mediator of cessation [42]. In the present study, we hypothesize that self-efficacy will both moderate and mediate outcome.

The Danaher et al. (2008) smoking trial identified program involvement as a second mediator. Participants who used program features more often, and completed more exercises, had better outcomes. A second Internet smoking cessation trial also found that program involvement mediated change [43]. Similarly, we hypothesize that degree of program involvement in the Self-Change Tools (SCT) intervention will mediate its increased `1`1`impact on reducing gambling behaviour compared with the control.

## Methods/Design

The proposed research will evaluate a full Internet-based self-directed intervention for problem gamblers using a single blind, randomized controlled trial comparing participants who are provided access to the SCTs as compared to those who are only provided the CYG personalized feedback screener (the control condition). There is one primary hypothesis and four secondary hypotheses:

Hypothesis 1**:** Respondents will display significant reductions in their gambling behaviour in the twelve months after being provided access to the online SCTs as compared to participants only provided with the normative feedback control intervention (CYG).

In addition, two potential moderators and two potential mediators of the impact of the full intervention, program involvement and perceived self-efficacy, will be tested as secondary hypotheses:

**Hypothesis 2:** Respondents with greater baseline self-efficacy will display significant reductions in their gambling behaviour in the twelve months after being provided access to the online SCTs or the CYG compared to participants with lower baseline self-efficacy.

**Hypothesis 3:** Respondents who have never accessed gambling or mental health treatment will display significant reductions in their gambling behaviour in the twelve months after being provided access to the online SCTs or the CYG compared to participants with previous treatment involvement.

**Hypothesis 4:** Respondents in the SCTs intervention condition who have more involvement with the SCTs intervention between baseline and three-month follow-up will demonstrate more improvement in gambling outcomes at six- and twelve-month follow-up, compared to respondents who have less involvement with the SCTs intervention.

**Hypothesis 5:** Respondents in the SCTs intervention condition will display significant increases in their perceived self-efficacy to deal with their gambling concerns in the three months after being provided access to the online SCTs as compared to participants in the control condition. Participants with greater improvement in self-efficacy will show greater improvement in gambling behaviour.

### Ethics approval

The research methods to be used in this study have been approved by the University of Calgary Conjoint Faculties Research Ethics Board (certificate 7279).

### Participant recruitment and randomization

Following procedures we have used in our earlier studies, media announcements (newspapers, radio, and websites) will be used to recruit individuals concerned about their gambling and interested in web-based self-directed treatment. Inclusion criteria will be identical to Hodgins et al. (2009): 18 years of age or older; perception of a gambling problem and scoring a 3 or greater on the Problem Gambling Severity Index of the Canadian Problem Gambling Index (PGSI- CPGI; [44]); gambled at least once in the past month; not involved in treatment at present (includes Gamblers Anonymous and any medical or psychological treatment where gambling problems are addressed); willingness and ability to access a website in English (to ensure reading ability); willingness to have telephone contacts recorded, willing to provide follow-up data on gambling; willingness to provide the name of a collateral (family or friend) to help locate them for follow-up interviews and the name of the same or a different collateral for data validation. Use of psychiatric medications for other mental health disorders will not be an exclusion criterion, although use will be assessed and monitored as a potential treatment moderator.

To ensure an adequate number of participants are assessed within the timeframe, recruitment will occur across Canada. Both urban and rural settings will be targeted. Interested individuals will be provided with a website address with information about the study, including eligibility criteria and a toll-free number to call or email address to use if interested. Having participants access a website at this point in the recruitment process is designed to minimize the number of participants who are randomly assigned but never access the intervention or control website (30% in one of our recent alcohol trials).

Individuals will be contacted by telephone and if they meet eligibility criteria and provide informed consent, they will receive a brief telephone assessment (gambling history and behaviour, self-efficacy) and then will be randomly assigned to one of the two intervention conditions, stratified on sex, gambling problem severity and treatment history (yes or no) [45] using MINIM, a computer program which uses the method of minimization. Problem severity, based on the NODS described below, will be defined as low-moderate (6 or fewer DSM-IV criteria) or high (7–10 criteria).

### Intervention conditions

#### Self-change Tools (SCTs)

SCTs, as described above, integrate the self-directed written materials [24] that were evaluated in the three brief treatment trials [13,15,46] and a trial of a self-directed relapse prevention for gambling problems [47]. A major focus is to provide individuals with clear and concise behavioural and cognitive strategies for meeting the goal of reducing or quitting gambling. The materials are presented as a series of options from which participants will choose what strategies seem most relevant to them. Participants will have ongoing access to the site over the follow-up period to make it as similar as possible to other web resources in terms of accessibility. The enhancements to the workbook content include three features. First, whereas the workbook encourages individuals to self-monitor their gambling, the website will provide a more structured option for individuals to maintain an online log of gambling and gambling urges. A further option will be to use a smartphone application to collect this self-monitoring information. A second enhancement is the expansion of the self-assessment component of the workbook to include more structured personalized normative feedback of gambling involvement. Again, the workbook provides some of this feedback but in a less comprehensive way. The third enhancement is the option for individuals to receive motivational email or text reminders of their progress and goals. This feature is somewhat similar to the motivational telephone booster calls provided in Hodgins et al. (2009).

#### Check your gambling (CYG; http://www.CheckYourGambling.net)

CYG will be used as the control intervention. Although we hypothesize that the SCTs will be a more powerful intervention than the CYG, CYG is a credible and ethically appropriate control comparison. In the CYG, the participant completes a brief assessment and then receives a personalized feedback report. The personalized feedback materials start out with a brief statement of the purpose of the report (“help to give you a picture of your gambling and let you know how your gambling compares with other Canadians”). The person is then provided with a summary of the types of gambling engaged in, along with a comparison of how this relates to other Canadians of their sex, a summary of their Problem Gambling Severity Index score and interpretation and a description of the types of gambling cognitions that the person endorsed. The final element of the feedback is a list of techniques that the person could adopt to lower the risk associated with their gambling.

### Baseline assessment

The brief telephone baseline assessment (adapted from Hodgins et al. 2009) will include a demographic profile (age, sex, education, marital status, income, ethnicity and racial status, employment status) and a gambling, mental health and treatment history including a timeline interview of types of gambling (online, land-based), frequency, and money spent for the past three months [48,49]. Problem gambling severity will be assessed using the past year Problem Gambling Severity Index and the lifetime and past three month version of the NORC DSM-IV Screen for Gambling Problems (NODS) [50,51] which indicates DSM-IV severity. Hodgins [52] administered the NODS to problem gamblers as part of a 1-year follow-up after a brief treatment to assess its utility as a treatment outcome measure. Internal reliability was fair to good and the factor structure and item-total correlations supported the existence of a single higher order construct that correlated moderately with gambling behavior and outcome. Self-efficacy will be administered using the Gambling Abstinence Self-efficacy Scale (GASS, [53], a 21 item self-report scale with evidence of concurrent and predictive validity in problem gambling treatment samples. In addition, participants will be asked to identify a treatment goal (quit or reduce gambling) and how successful they think they would be (0 “not at all” to 10 “extremely”) in the next 6 months and in the next 12 months. The Kessler 10 (K10) questionnaire will be included to provide a continuous measure of general psychological distress that is responsive to change over time. The K10 has been well validated and its brevity and simple response format are attractive features. It also produces a summary measure indicating probability of currently experiencing an anxiety or depressive disorder [54,55]. Quality of life will be assessed by the WHOQoL-8, an eight item version of a widely used measure. This short form has been used in a number of countries, is robust psychometrically, and overall performance is strongly correlated with scores from the original WHOQoL [56].

### Follow-up assessment

After three, six and twelve months, a follow-up assessment of gambling behaviour (timeline method), problem gambling severity (NODS), self-rated improvement, self-efficacy (GASS), psychiatric distress, quality of life, use of other treatment resources, and impressions of site features and tools will be conducted. Although it would appear efficient to conduct follow-up assessments via the Internet, previous experience has shown that attrition is extremely high with this type of design. In our previous research, follow-up interviews were conducted via telephone with good follow-up rates. As a result, our follow-up interviews in this study will be conducted via telephone. We will collect extensive contact information for participants to minimize losing contact (e.g., contact information for family and friends, work contact, email contact, etc.).

Participants will also be asked to provide the name of a collateral (family or friend) who can confirm their self-reported gambling behaviour through a brief telephone call. In Hodgins et al., (2001, 2009) agreement between participants and collaterals was r = .61 and ICC = .69 for dollars lost and r = .66 and ICC = .53 for days of gambling, indicating fair agreement. As in previous research [49,57], collaterals who described themselves as more confident showed better agreement and in general collaterals tended to report less gambling than participants suggesting that participant reports are not attenuated by minimization.

### Process measures

We will have access to a complete record of the amount and type of use participants make of the SCTs (and the CYG). Following methods used by Danaher (2008) and Strecher (2008), we will operationalize degree of involvement with SCTs by recording the number of times the participant accesses the site as well as the number of tools the participant uses (as assessed by page views, form completions, etc.) and length of involvement with the site (e.g. use of the site over time). This information will be used to test the mediation hypothesis that degree of involvement is related to success at overcoming gambling problems.

### Primary and secondary outcome variables

Two major outcome variables for testing the primary and secondary hypotheses will be: mean days per month gambling and NODS scores. Mean dollars lost per gambling day, total dollars lost and self-rated improvement will be used as secondary outcome variables. Days and dollars spent gambling will be calculated for the three months pre-treatment and for each follow-up period. The data will be inspected for approximate normality or symmetry and, if necessary, subjected to appropriate transformation.

### Blinding

Participants will be aware that they will be assigned to one of two intervention options, although neither one will be described as potentially superior. Baseline assessment occurs prior to randomization. Follow-up assessments are conducted by interviewers who will be blind to participant assignment.

### Analyses plan

Primary analyses for Hypothesis 1, comparing outcomes for the two groups, will be based on the appropriate random effects model to properly account for the longitudinal nature of this data. Condition (2) and Time (0,3,6,12) will be modeled as fixed factors while the participants will be modeled as random factors. Separate analyses will be conducted for each primary and secondary outcome variable. This same analytic approach will be used for Hypothesis 2 and 3, examining moderators. Baseline self-efficacy, past use of treatment, and baseline NODS scores will be included as additional factors. For Hypothesis 3 and 4, the mediation hypotheses, the procedures recommended by Preacher and Hayes [58] will be conducted using a series of random effect models. Missing data should not be a major issue but techniques for these matters may be used depending on the nature, location and type of missingness.

### Sample size and power analysis

We propose to collect a sample of 180 participants and we estimate (based upon Hodgins et al., 2009) that we will successfully follow about 153 participants at three months. This number will ensure a heterogeneous sample of individuals that will provide a valid assessment of the perceived value of different components of the SCTs. Based upon previous experience, about half will be of each sex. This number will also provide sufficient power to conduct the proposed statistical tests comparing the two conditions, based upon gambling frequency and NODS data from Hodgins et al. (2001, 2009), assuming a correlation of .5 between baseline and follow-up values, power = 0.80 and a Bonferroni corrected α = .025 (.05/2 outcome variables). This sample size is estimated so as to be sufficient to detect a difference of about 2 gambling days per month between conditions at each follow-up interval (medium effect size). This degree of difference is clinically meaningful in terms of gambling involvement. Similarly, this sample size will detect a 1 point difference on the NODS at 12 months. Given the complexity of estimating power for HLM models, these calculations are based upon a more simple repeated measures ANOVA model [59], with an attrition rate (i.e., not followed after baseline assessment) estimated to be 15%. The proposed hlm analysis will likely have greater statistical power because all observed data are included.

## Discussion

The goal of this proposal is to develop an evidence-based web resource to provide help to problem gamblers. There is a great deal of interest for such a resource in a variety of jurisdictions including a number of Canadian provinces.

The design has a number of limitations. Our recruitment method and telephone follow-up is designed to maximize the retention of participants in the trial (i.e., enhance the internal validity of the research). This design may limit the generalizability of the findings (i.e., external validity) as we will not assess the efficacy with individuals who are unwilling to enter a clinical trial but would who briefly visit a site and look at or use one or more tools. Future research will be required to address the issue of how effective a free online resource would be. A second limitation of the design is that we will not be able to isolate the effective ingredients of the intervention as numerous strategies are available for use. Protocols for outcome studies of face-to-face therapies ensure that adherent participants (i.e., those that attend sessions) are exposed to all active treatment components. In contrast, with self-directed treatment, these components are offered as a menu of options. “Adherent” participants (i.e., those that visit the website) may select only a few strategies. However, we will be tracking the use of the various program components so we will be able to conduct some correlational analysis of use and outcome to develop hypotheses for future research. Similarly, although we will recruit individuals who are not currently accessing treatment for gambling problems, individuals may choose to access treatment resources during the trial. In fact, as we have in previous trials, we will provide information about treatment resources to all participants. We will monitor involvement and describe this involvement at each follow-up (overall very low in previous trials).

Treatment outcome research relies on self-reported gambling behaviour. We will collect this information using standardized and validated procedures to enhance accuracy and we will also confirm using collateral reports from family and friends. Nonetheless, reliance on self-report adds variability to the data. A final limitation is the relatively brief assessment of participants. In order to minimize the potential therapeutic impact of the assessment process, we desire to keep it brief but we also want to explore potential predictors of outcome. Therefore, a number of assessment domains (e.g. comorbid mental health, social support, quality of life) are assessed using very brief instruments and the extensive data required to examine cost effectiveness will not be collected in this trial. Future, more targeted research with comprehensive instruments will be required to follow-up on positive results.

Despite these limitations, this trial has many design strengths which reinforce the importance of the results to policy-makers and treatment service administrators. Having numerous entryways into treatment and recovery and many types of treatment and treatment supports are crucial in effectively tackling this significant problem.

## Competing interests

The authors declare that they have no competing interest. Dr. Hodgins has received consulting fees from the Centre for Addiction and Mental Health for development of the online gambling tool.

## Authors’ contributions

All authors have made an intellectual contribution to this project. DH has overall responsibility for the trial. DH and JC conceived and designed the project. GF provided statistical analysis and design consultation. RM conceived and developed the online tool. All authors contributed to the protocol. All authors read and approved the final manuscript.

## Pre-publication history

The pre-publication history for this paper can be accessed here:

http://www.biomedcentral.com/1471-2458/13/10/prepub
